# Machine learning-based COVID-19 prognostic models lag behind in reporting quality: findings from a TRIPOD/TRIPOD + AI systematic review

**DOI:** 10.1186/s41512-026-00218-x

**Published:** 2026-02-03

**Authors:** Ioannis Partheniadis, Persefoni Talimtzi, Adriani Nikolakopoulou, Anna-Bettina Haidich

**Affiliations:** 1 Laboratory of Pharmaceutical Technology, School of Pharmacy, Faculty of Health Sciences, Aristotle University of Thessaloniki, University Campus, Thessaloniki, 54124 Greece; 2 Department of Hygiene, Social-Preventive Medicine and Medical Statistics, School of Medicine, Faculty of Health Sciences, Aristotle University of Thessaloniki, University Campus, Thessaloniki, 54124 Greece; 3 Institute of Medical Biometry and Statistics, Faculty of Medicine and Medical Center, University of Freiburg, Freiburg, Germany

**Keywords:** Prognostic models, COVID-19, Reporting completeness, Machine learning, TRIPOD, TRIPOD + AI

## Abstract

**Background:**

Reporting of COVID-19 prognostic models frequently falls short of established standards. The TRIPOD checklist and its 2024 AI extension (TRIPOD + AI) provide a comprehensive framework for assessing reporting quality. We therefore evaluated and compared reporting completeness in conventional versus machine-learning models.

**Methods:**

Studies reporting the development, and internal and external validation of prognostic prediction models for COVID-19 using either conventional or machine learning-based algorithms were included. Literature searches were conducted in MEDLINE, Epistemonikos.org, and Scopus (up to July 31, 2024). Studies using conventional statistical methods were evaluated under TRIPOD, while machine learning-based studies were assessed using TRIPOD + AI. Data extraction followed TRIPOD and TRIPOD + AI checklists, measuring adherence per article and per checklist item. The protocol was prospectively registered at the Open Science Framework (https://osf.io/kg9yw).

**Results:**

A total of 53 studies describing 71 prognostic models were identified. Overall, adherence to both guidelines was low, with significantly poorer compliance among machine learning-based studies (TRIPOD + AI) compared to conventional model studies (TRIPOD) (28.4% vs. 38.1%, 95% CI of difference: 4.1–15.4). No study fully adhered to abstract reporting requirements, and appropriate titles were included in only a minority of cases (29.0%, 95% CI: 16.1–46.6 for TRIPOD; 13.6%, 95% CI: 4.8–33.3 for TRIPOD + AI). Sample size calculations were not fully reported in any study. Reporting of methods and results sections was poor across both frameworks.

**Conclusion:**

Lower adherence among machine learning studies reflects the relatively recent publication of the TRIPOD + AI guidelines (April 2024), which postdate many of the included studies. Both conventional and machine learning-based prediction models showed insufficient reporting, with major gaps in model description and performance reporting. Greater compliance with reporting guidelines is critical to improving the clarity, reproducibility, and clinical value of prediction model research.

**Supplementary Information:**

The online version contains supplementary material available at 10.1186/s41512-026-00218-x.

## Introduction

COVID-19, while no longer designated a public health emergency of international concern, continues to pose a major global health challenge [1]. As all countries have eased restrictions, concerns remain about the long-term behavior of SARS-CoV-2, which may become endemic with recurring seasonal surges that could burden healthcare systems [2]. The unpredictable nature of viral mutations, along with gaps in surveillance and response mechanisms, highlights the potential for future outbreaks [3]. In this context, accurate prognostic modeling plays a vital role in guiding clinical decisions, optimizing resource distribution, and shaping public health strategies. In response, the research community has produced a wide array of predictive models to estimate the risk of severe outcomes among individuals infected with COVID-19 [4]. These models are especially important in resource-constrained settings, where prioritizing care for the most at-risk patients is essential. Given the unprecedented volume of scientific data generated during the pandemic, critically evaluating and synthesizing this body of work remains a pressing need.

A major obstacle to the effective use and evaluation of clinical prediction models is the incomplete reporting of critical methodological and outcome-related information [5, 6]. Insufficient transparency not only hampers the ability to assess how a study was conducted or interpret its findings but also limits opportunities for independent validation by other researchers [7, 8]. Systematic reviews serve as a key bridge between model development and clinical use by aggregating and critically appraising available evidence, identifying promising models, and informing the local validation efforts necessary for safe and effective implementation. However, poor reporting undermines efforts to include such studies in systematic reviews and, in turn, restricts their translation into clinical practice. As a result, it contributes to research inefficiency and delays the integration of valuable tools into healthcare decision-making.

The Transparent Reporting of a multivariable prediction model for Individual Prognosis Or Diagnosis (TRIPOD) statement was introduced to enhance the quality and transparency of clinical prediction model reporting, offering a 22-item checklist with 37 sub-items that specify the essential elements for publishing studies on the development and validation of diagnostic or prognostic models, primarily those based on regression techniques [9]. Since its release, the use of machine-learning approaches to build clinical prediction models has surged, particularly amid the COVID-19 pandemic, as larger datasets and more accessible software have lowered the barrier to model development even when rigorous validation is lacking [10]; nevertheless, there is limited research assessing how comprehensively such machine learning-based prediction models are reported in the literature [11].

While many key reporting elements (study dates, sample size) are relevant to both regression-based and machine learning–based prediction model studies, it cannot be assumed that the reporting quality and methodological practices are consistent across these approaches. There is limited understanding of the specific reporting challenges associated with machine learning models and how these differ from those seen in traditional regression models [12]. In response to these challenges, an updated guideline (TRIPOD + AI) has been introduced to specifically address the reporting standards for machine learning-based prediction models, offering a structured framework for evaluating their completeness [13].

The present work offers a critical assessment of the reporting completeness of prognostic models developed using either conventional statistical or machine learning methods, with a focus on models reporting development, internal and external validation. It evaluates the extent to which these models adhere to the TRIPOD and TRIPOD + AI reporting guidelines, providing a basis for reflecting on the transparency of reporting practices in prognostic model studies. While TRIPOD + AI supersedes TRIPOD, it introduces AI-specific reporting requirements that do not apply to traditional regression-based models. Given the retrospective nature of this work, and the fact that many included studies were published before the release of TRIPOD + AI, using both guidelines enables a more balanced and historically grounded assessment of model reporting, without penalizing either group for failing to report items irrelevant to their methodology. This dual-framework approach also offers a unique opportunity to explore how evolving reporting standards affect the evaluation of models developed under different methodological paradigms and timeframes. The findings may contribute valuable insights for enhancing the quality and transparency of prognostic model reporting in COVID-19 research.

## Materials and methods

### Protocol and registration

This study was prospectively registered with a predefined protocol at the Open Science Framework (OSF), accessible at https://osf.io/kg9yw. Despite being methodological in nature, the reporting of the study follows the principles of the Preferred Reporting Items for Systematic Reviews and Meta-Analyses (PRISMA) 2020 statement [14] (Appendix Table 1), when applicable.

### Eligibility criteria

This study includes prognostic model studies for COVID-19 reporting model development, internal validation, and external validation, as identified through systematic reviews (SRs) that met predefined eligibility criteria. These SRs served as sources for identifying eligible primary studies and were selected based on the criteria defined by Talimitzi et al. [15]. Briefly, reviews were considered eligible if they explicitly identified themselves as SRs, outlined a predefined research question, and clearly specified both the scope of the review and the inclusion criteria for studies. Only SRs published in English were included. Exclusion criteria encompassed reviews centered on epidemic forecasting models, diagnostic test accuracy, randomized controlled trials comparing COVID-19 treatments, methodological and genetic association reviews, as well as letters to the editor and commentaries. Within each included review, we selected prognostic model studies if they reported all following elements: model development, internal validation, and external validation. Studies lacking any of these components were excluded. This criterion was chosen to ensure that reporting completeness could be assessed across the full modeling process, from development to external validation, which is critical for evaluating model readiness for clinical application.

### Databases and search strategy

The literature search was conducted using the following bibliographic databases: MEDLINE (via Ovid), Scopus, the Cochrane Database of Systematic Reviews, and Epistemonikos (epistemonikos.org), covering publications up to July 31, 2024. In addition, a citation chasing approach, both backward and forward referencing, was applied using the ‘citationchaser’ package in R [16] to identify related studies cited by or citing the included articles. The search strategy (Appendix Table 2) followed the methodology proposed by Talimtzi et al. [15]., which was an adaptation of the approach used by Wynants et al. [10]., with the goal of capturing all SRs focused on COVID-19 prognostic models.

### Screening and selection of studies

Data was extracted for each prognostic model identified within the included systematic reviews using structured forms in Microsoft Excel^®^. Duplicate records were removed, after which one reviewer (I.P.) screened the titles and abstracts of the remaining articles. Full-text reviews were conducted for all potentially relevant studies by one reviewer (I.P.), and those that met the inclusion criteria were incorporated into the present study.

### Data extraction

One reviewer (I.P.) carried out data extraction for all included studies after reaching > 90% agreement with a second reviewer (P.T.) who independently extracted data from a randomly selected 10% subset of the included studies. Any discrepancies between the two reviewers were resolved through discussion with a third reviewer (A.B.H.). For each eligible study, information such as the first author, journal, publication year, and additional methodological characteristics, as guided by the CHARMS checklist [17] and listed in Appendix Table 3, was recorded. When a study reported multiple models but identified one as the primary or “best” model, only data for that specific model were extracted. Conversely, if a study aimed to develop or present multiple models without prioritizing one, data were extracted for each model individually. Briefly, data extraction focused on model-specific details aligned with TRIPOD/TRIPOD + AI guidelines, including: target population and setting, participant characteristics, sample size, handling of missing data, study design and model development process, model performance, internal and external validation, and final model presentation. Transparency indicators were evaluated at both the study and journal levels. At the journal level, we extracted whether the journal in which each study was published had explicit policies on data sharing, code availability, and adherence to TRIPOD (Table 1).

**Table 1:** Characteristics of the included studies

Characteristics on Study Level (*n* = 53)	
Publication Year	N (%)
2020	27 (50.9)
2021	23 (43.4)
2022	2 (3.8)
2023	1 (1.9)
Preprint only	N (%)
Yes	3 (5.7)
No	50 (94.3)
Number of Models	N (%)
1	44 (83.0)
2	6 (11.3)
3	1 (1.9)
4	1 (1.9)
8	1 (1.9)
Study Design (Development & Internal Validation)	N (%)
Retrospective	44 (83.0)
Unclear	5 (9.4)
Prospective	3 (5.7)
Temporal Validation	1 (1.9)
Published in Journals with Code/Data Availability Policy	N (%)
Yes (does not specify for code)	23 (43.4)
Yes (both data and code)	21 (39.6)
No	9 (17.0)
Reporting Adherence to TRIPOD Statement	N (%)
Yes	16 (30.2)
No	37 (69.8)
TRIPOD Checklist Provided	N (%)
Yes	7 (13.2)
No	46 (86.8)
Published in Journals Suggesting TRIPOD Guidelines	N (%)
Yes	4 (7.5)
No	49 (92.5)

### TRIPOD and TRIPOD + AI adherence assessment

The adherence assessment approach followed the methodology outlined on the official TRIPOD statement website [18], as well as the guidance provided in the TRIPOD + AI statement [13] for models developed using supervised machine learning, and was consistent with the approach used in a previously published study [11, 19]. Applying both guidelines selectively avoided penalizing studies for omitting information not relevant to their modeling approach. For the purpose of this study, we categorized each prognostic model as either conventional (statistical) or machine learning–based, based on the methodology used. Models using traditional regression techniques (e.g., logistic regression, Cox regression) were assessed using the TRIPOD checklist, while models employing machine learning methods (e.g., random forests, neural networks, gradient boosting) were assessed using the TRIPOD + AI checklist. In cases where a single study reported both model types, each model was evaluated individually according to the appropriate guideline. Adherence was evaluated for each individual TRIPOD/TRIPOD + AI item. If all relevant adherence elements for a specific item were marked as “yes” or “Not Applicable,” the item was considered reported and assigned a score of “1”; otherwise, it was scored as “0.” An overall adherence score for each report was then calculated by dividing the number of reported items by the total number of applicable items. Each TRIPOD or TRIPOD + AI item was treated as a distinct reporting element with individual interpretive value; therefore, we conducted unadjusted item-level comparisons without applying corrections for multiple testing, consistent with the checklist-based structure of the guidelines. In addition, overall adherence for each individual item was determined by dividing the number of studies that adhered to that item by the number of studies for which that item was applicable.

### Summary measures and synthesis of results

The results are presented using descriptive statistics, visualizations, and narrative summaries. For continuous variables, data is reported as mean and standard deviation (SD) when normally distributed, or as median and interquartile range (IQR) when distributions are non-normal. Categorical variables are summarized as frequencies and percentages. For each study, we calculated the events-per-predictor ratio by dividing the total number of observed outcome events by the number of candidate predictors initially considered, regardless of whether they were retained in the final model. While not a definitive measure of sample size adequacy, this ratio offers a heuristic indication of potential overfitting risk, with lower values generally associated with reduced model stability and reliability.

To explore differences in methodological practices between conventional and machine learning–based prediction models, adherence to the TRIPOD and TRIPOD + AI guidelines was evaluated. Comparisons are conducted on two levels: (a) adherence to each (sub-)item within the TRIPOD/TRIPOD + AI checklists, and (b) the overall adherence score for each study, representing the reporting quality of each developed model. Chi-square tests or Fisher’s exact tests (when expected cell counts were fewer than five) were used to compare adherence frequencies between statistical and machine learning models. Odds ratios (ORs) with 95% confidence intervals (CIs) were calculated to quantify the strength of association between model type and adherence to each individual reporting item. Mean differences in overall adherence between the two model types, along with their 95% confidence intervals (CIs), were assessed using independent-samples t-tests, as appropriate based on data distribution.

All statistical analyses were carried out using R (version 4.4.0; R Foundation for Statistical Computing, Vienna, Austria) and RStudio (version 2024.9.0.375).

## Results

### Study selection

**Fig. 1:**
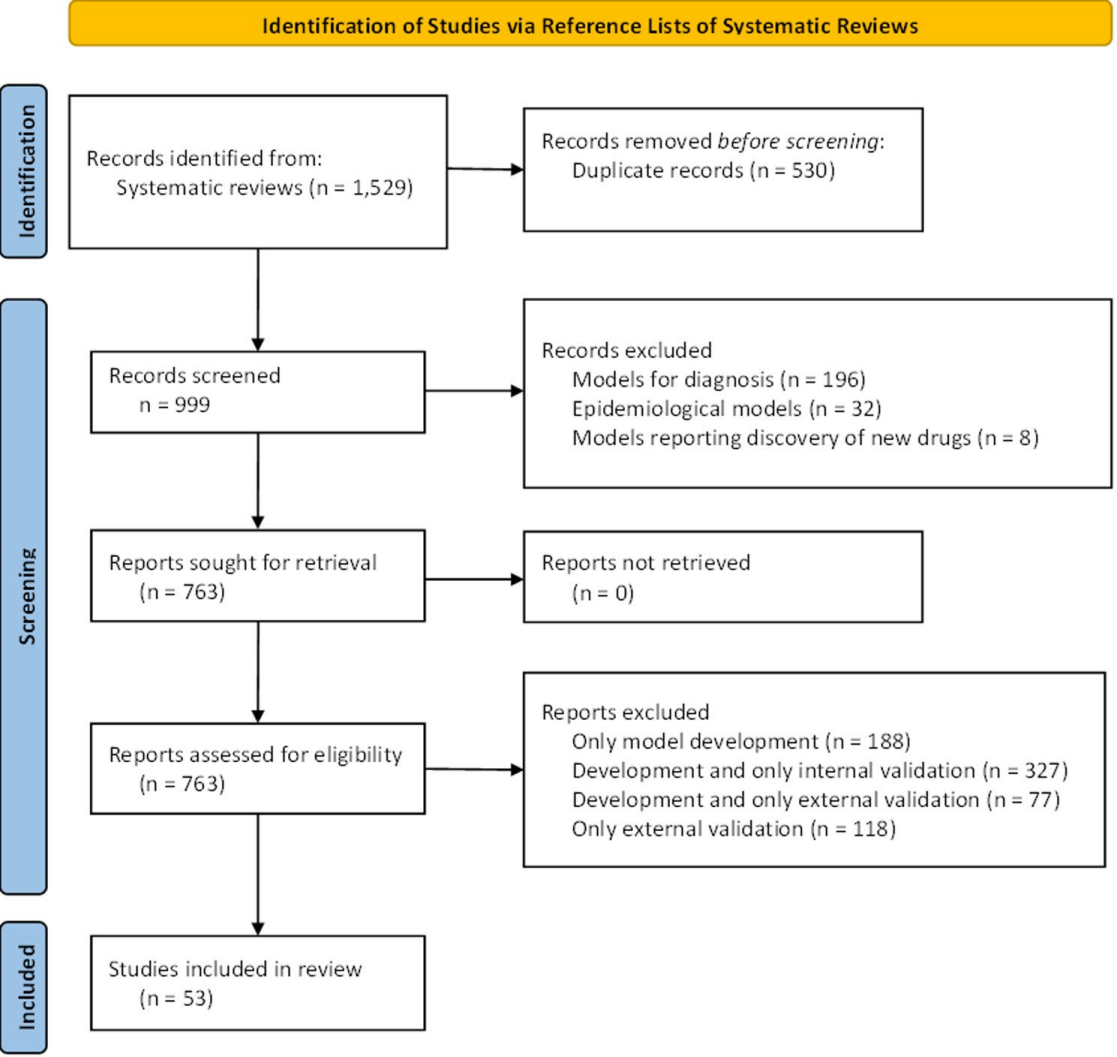
PRISMA flowchart of the study selection process

A total of 1,529 records were retrieved from 17 SRs and initially screened by title and abstract. Following the removal of 530 duplicates, 999 studies proceeded to full-text review. Of these, 53 studies met the predefined eligibility criteria, collectively reporting 71 prognostic models that included details on model development, as well as internal and external validation. A complete list of the 17 SRs initially considered as sources, along with the studies ultimately included, can be found in the Appendix (Appendix Tables 4 and 5). The PRISMA flow diagram outlining the study selection process is presented in Fig. 1.

### Characteristics of the included studies

Table 1 summarizes the characteristics of the included studies, including publication year, study design, number of models, and journal-level transparency policies. Most were published in 2020 (50.9%) and 2021 (43.4%), with the vast majority (94.3%) appearing in peer-reviewed journals. A single final model was reported in 83.0% of studies. Regarding study design for development and internal validation, most studies (83.0%) were retrospective in nature, while only a small proportion were prospective (5.7%) or used temporally split data (1.9%). In 9.4% of cases, the design was not clearly described. Journal-level transparency policies were relatively common: 83.0% of the journals had data or code availability requirements, although not all specified both elements. Specifically, 39.6% required both data and code availability, while 43.4% mentioned only data. However, only 30.2% of the studies themselves explicitly reported adherence to the TRIPOD Statement, and just 13.2% included a completed checklist as supplementary material. A small minority of journals (7.5%) explicitly recommended using TRIPOD in their author instructions.

### Characteristics of the included models

Table 2 summarizes the characteristics of the 71 included models, which were nearly evenly split, with 58.5% being traditional statistical models and 41.5% being machine learning-based models. Common predictors included age (67.6%), C-reactive protein and lactic dehydrogenase (both 32.4%), lymphocyte count (29.6%), and oxygen saturation (29.6%). The most frequently predicted outcomes were in-hospital mortality (43.7%) and severe or critical illness (38.0%). Most models (71.8%) were based on single-country data, with China (50.7%) and the U.S. (26.8%) being the most common sources The models predominantly focused on hospitalized patients (87.3%) with confirmed COVID-19 (94.4%). Recruitment methods were often unclear (52.1%), though 47.9% used consecutive sampling. Most studies used multicenter data (87.3%), with few relying on single-center (8.5%) or nationwide cohorts (4.2%). Based on 64 models, the median participant age was 61 years (IQR: 58–63), and the average proportion of male participants was 54.8% (SD: 6.6%). Seven models did not report participant age and were therefore excluded from this summary.

**Table 2:** Characteristics of the included models

Characteristics on Model Level (*n*= 71)	
Type of Models	N (%)
Conventional	36 (50.7)
Machine learning	35 (49.3)
Most Common Predictors	N (%)
Age	48 (67.6)
C-reactive protein	23 (32.4)
Lactic dehydrogenase	23 (32.4)
Lymphocytes	21 (29.6)
Oxygen saturation	21 (29.6)
D-dimer	12 (16.9)
Neutrophils	12 (16.9)
Platelets	11 (15.5)
Sex	11 (15.5)
Blood urea nitrogen	10 (14.1)
Most Common Outcomes	N (%)
Mortality	31 (43.7)
Critical/severe illness	27 (38.0)
Single Country	N (%)
Yes	51 (71.8)
No	16 (22.6)
Unknown	4 (5.6)
Countries of Setting/Center Location (most common)	N (%)
China	36 (50.7)
USA	19 (26.8)
Italy	6 (8.5)
UK	6 (8.5)
France	4 (5.6)
Spain	4 (5.6)
Iran	2 (2.8)
Kuwait	2 (2.8)
Target Population	N (%)
Hospitalized patients	62 (87.3)
Other a	5 (7.1)
Unclear	2 (2.8)
Outpatients	1 (1.4)
Patients at triage center	1 (1.4)
Population’s COVID-19 Status	N (%)
Confirmed cases	67 (94.4)
Unclear	3 (4.2)
Confirmed or suspected cases	1 (1.4)
Characteristics on Model Level (*n*= 71)	
Recruitment Method (Development & Internal Validation)	N (%)
Unclear	37 (52.1)
Consecutive	34 (47.9)
Recruitment Center	N (%)
Multicenter	62 (87.3)
Monocenter	6 (8.5)
Nationwide	3 (4.2)
Average Age Reported, *years, median (IQR)*	61.00 (58.00–63.00)
Not reported, N (%)	7 (9.8)
Sex, *% male, mean (SD)*	54.79 (6.58)
Not reported, N (%)	5 (7.1)
C-statistic (Concordance Index) (Internal Validation), mean (SD)	0.86 (15.63)
Not reported, N (%)	8 (11.3)
Sensitivity (Internal Validation), %, median (IQR)	73.50 (53.50–89.00)
Not reported, N (%)	41 (57.7)
Specificity (Internal Validation), %, median (IQR)	85.00 (77.00–89.00)
Not reported, N (%)	42 (59.2)
C-statistic (Concordance Index) (External Validation), mean (SD)	0.84 (0.09)
Not reported, N (%)	4 (5.6)
Sensitivity (External Validation), %, median (IQR)	76.00 (67.00–86.25)
Not reported, N (%)	39 (54.9)
Specificity (External Validation), %, median (IQR)	82.00 (73.00–92.25)
Not reported, N (%)	39 (54.9)

^a^Other: all individuals undergoing a SARS-CoV-2 test, outpatients with diabetes, Elderly COVID-19 patients, In- and out-patients

### Per item adherence to TRIPOD and TRIPOD + AI statements

Table 3 presents item-level adherence to the TRIPOD and TRIPOD + AI guidelines, along with ORs and corresponding 95% CIs comparing adherence between the two frameworks. Conventional model studies were assessed using TRIPOD, and machine learning model studies using TRIPOD + AI. Items are aligned by section/topic to facilitate meaningful comparisons, while preserving methodological appropriateness. A comprehensive list of the methodological characteristics of the included studies that were used for adherence evaluation can be found in Appendix Table 6.

However, the study has limitations. Reporting quality was assessed by a single reviewer, which may introduce bias although there was a good agreement in the subsample that was assessed independently by a second reviewer. Also, only the best-performing model from each study was evaluated, potentially underestimating broader reporting deficiencies. In addition, different reporting frameworks were applied to conventional and machine-learning models (TRIPOD and TRIPOD + AI, respectively). Because TRIPOD + AI contains several items that extend beyond AI-specific considerations and have no direct counterparts in TRIPOD, this discrepancy in checklist scope may influence overall adherence estimates. We therefore presented item-level adherence for both guidelines to clarify how individual items contributed to observed differences (Table 3), but comparisons of overall adherence should be interpreted cautiously in light of the structural differences between the two checklists. Lastly, while reporting guidelines aim to standardize transparency, they are not formal tools for evaluating study quality, which may affect interpretation.

**Table 3:** Calculated % adherence per item to the TRIPOD and TRIPOD+AI (studies adhered to item/studies in which item was applicable) and odds ratio for the comparison TRIPOD *vs*. TRIPOD+AI together with 95% confidence intervals (n.a. stands for not applicable; n.e. stands for not estimated). Items are grouped by reporting section/topic to ensure interpretability across guidelines with differing scopes

Section/Topic	Item		TRIPOD Item Adherence (%)	95% CI	TRIPOD+AI Item Adherence (%)	95% CI	TRIPOD *vs*. TRIPOD+AI OR (95% CI)	*p*-value
	TRIPOD	TRIPOD+AI						
Title	1	1	29.0 (9/31)	16.1–46.6	13.6 (3/22)	4.8–33.3	2.55 (0.53–16.74)	0.3182
Abstract	2	2	0.0 (0/31)	0.0–11.0	0.0 (0/22)	0.0–14.9	n.a.	n.a.
Introduction: Background	3a	3a	48.4 (15/31)	32.0–65.2	40.9 (9/22)	23.3–61.3	1.35 (0.45–4.08)	0.7957
	n.a.	3b	n.a.	n.a.	9.1 (2/22)	2.5–27.8	n.a.	n.a.
	n.a.	3c	n.a.	n.a.	n.a.	n.a.	n.a.	n.a.
Introduction: Objectives	3b	4	67.7 (21/31)	50.1–81.4	45.5 (10/22)	26.9–65.3	2.52 (0.82–7.78)	0.1804
Methods: Source of Data	4a	5a	90.3 (28/31)	75.1–96.7	22.7 (5/22)	22.7–43.4	31.73 (6.72–149.97)	< 0.0001
	4b	5b	32.3 (10/31)	18.6–49.9	13.6 (3/22)	5.5–37.6	3.02 (0.72–12.62)	0.2192
Methods: Participants	5a	6a	90.3 (28/31)	75.1–96.7	72.7 (16/22)	51.8–86.9	3.41 (0.62–24.02)	0.1396
	5b	6b	41.9 (13/31)	26.4–59.2	63.6 (14/22)	43.0–80.3	0.41 (0.13–1.27)	0.2011
	5c	6c	n.a.	n.a.	n.a.	n.a.	n.a.	n.a.
Methods: Data Preparation	n.a.	7	n.a.	n.a.	13.6 (3/22)	4.8–33.3	n.a.	n.a.
Methods: Outcome	6a	8a	29.0 (9/31)	16.1–46.6	36.4 (8/22)	19.7–57.1	0.72 (0.22–2.29)	0.7912
	n.a.	8b	n.a.	n.a.	0.0 (0/1)	0.00–79.4	n.a.	n.a.
	6b	8c	45.2 (14/31)	29.2–62.2	40.9 (9/22)	23.3–61.3	1.19 (0.39–3.59)	0.9788
Methods: Predictors	10b	9a	6.5 (2/31)	1.8–20.7	50.0 (11/22)	30.7–69.3	0.07 (0.01–0.41)	< 0.0001
	7a	9b	6.5 (2/31)	1.8–20.7	9.1 (2/22)	2.5–27.8	0.69 (0.05–10.32)	0.9999
	7b	9b	0.0 (0/31)	0.0–11.0	9.1 (2/22)	2.5–27.8	0.00 (0.00–3.73)	0.1676
	n.a.	9c	n.a.	n.a.	0.0 (0/2)	0.0–65.8	n.a.	n.a.
Methods: Sample Size	8	10	0.0 (0/31)	0.0–11.0	0.0 (0/22)	0.0–14.9	n.a.	n.a.
Methods: Missing Data	9	11	35.5 (11/31)	21.1–53.1	9.1 (2/22)	2.5–27.8	5.50 (1.08–28.05)	0.0606
Methods: Analysis	n.a.	12a	n.a.	n.a.	4.6 (1/22)	0.8–21.8	n.a.	n.a.
	10a	12b	19.4 (6/31)	9.2–36.3	40.0 (6/15)	19.8–64.3	0.36 (0.09–1.41)	0.1649
	10b	12c	6.5 (2/31)	1.8–20.7	4.6 (1/22)	0.8–21.8	1.44 (0.07–89.41)	0.9999
	n.a.	12d	n.a.	n.a.	n.a.	n.a.	n.a.	n.a.
	10c	12g	16.1 (5/31)	7.1–32.6	36.4 (8/22)	19.7–57.1	0.34 (0.09–1.23)	0.1729
	10d	12e	48.4 (15/31)	32.0–65.2	0.0 (0/22)	0.0–14.9	n.e.	n.e.
	10e	12f	n.a.	n.a.	n.a.	n.a.	n.a.	n.a.
Methods: Risk groups	11	n.a.	64.3 (9/14)^a^	38.8–83.7	n.a.	n.a.	n.a.	n.a.
Methods: Model Output	n.a.	15	n.a.	n.a.	68.2 (15/22)	47.3–83.6	n.a.	n.a.
Methods: Class imbalance	n.a.	13	n.a.	n.a.	100.0 (1/1)^b^	20.7–100.0	n.a.	n.a.
Methods: Fairness	n.a.	14	n.a.	n.a.	n.a.	n.a.	n.a.	n.a.
Methods: Development vs. Validation	12	16	3.2 (1/31)	0.6–16.2	9.1 (2/22)	2.5–27.8	0.34 (0.01–6.95)	0.5633
Methods: Ethical Approval	n.a.	17	n.a.	n.a.	95.5 (21/22)	78.2–99.2	n.a.	n.a.
Open Science: Funding	22	18a	41.9 (13/31)	26.4–59.2	27.3 (6/22)	13.2–48.2	1.93 (0.59–6.26)	0.4202
Open Science: Conflicts of interest	21	18b	74.2 (23/31)	56.8–86.3	68.2 (15/22)	47.3–83.6	1.34 (0.40–4.48)	0.8656
Open Science: Protocol	21	18c	74.2 (23/31)	56.8–86.3	0.0 (0/22)	0.0–14.9	n.e.	n.e.
Open Science: Registration	21	18d	74.2 (23/31)	56.8–86.3	13.6 (3/22)	4.8–33.3	18.21 (4.23–78.36)	< 0.0001
Open Science: Data Sharing	21	18e	74.2 (23/31)	56.8–86.3	31.8 (7/22)	16.4–52.7	6.16 (1.85–20.56)	0.0053
Open Science: Code Sharing	21	18f	74.2 (23/31)	56.8–86.3	31.8 (7/22)	16.4–52.7	6.16 (1.85–20.56)	0.0053
Patient and Public Involvement	n.a.	19	n.a.	n.a.	13.6 (3/22)	4.8–33.3	n.a.	n.a.
Results: Participants	13a	20a	6.5 (2/31)	1.8–20.7	4.6 (1/22)	0.8–21.8	1.44 (0.07–89.41)	0.9999
	13b	20b	19.4 (6/31)	9.2–36.3	63.6 (14/22)	43.0–80.3	0.14 (0.04–0.48)	0.0028
	13c	20c	54.8 (17/31)	37.8–70.8	n.a.	n.a.	n.a.	n.a.
Results: Model Development	14a	21	32.3 (10/31)	18.6–49.9	40.9 (9/22)	23.3–61.3	0.69 (0.22–2.14)	0.7215
	14b	n.a.	70.6 (12/17)	46.9–86.7	n.a.	n.a.	n.a.	n.a.
Results: Model Specification	15a	22	12.9 (4/31)	5.1–28.9	4.6 (1/22)	0.8–21.8	3.05 (0.28–160.25)	0.3886
	15b	n.a.	54.8 (17/31)	37.8–70.8	n.a.	n.a.	n.a.	n.a.
Results: Model Performance	16	23a	16.1 (5/31)	7.1–32.6	0.0 (0/22)	0.0–14.9	n.e.	n.e.
	n.a.	23b	n.a.	n.a.	n.a.	n.a.	n.a.	n.a.
Results: Model Updating	17	24	n.a.	n.a.	n.a.	n.a.	n.a.	n.a.
Discussion: Interpretation	19a	n.a.	41.9 (13/31)	26.4–59.2	n.a.	n.a.	n.a.	n.a.
	19b	25	96.8 (30/31)	83.8–99.4	0.0 (0/22)	0.0–14.9	n.e.	n.e.
Discussion: Limitations	18	26	93.6 (29/31)	79.3–98.2	86.4 (19/22)	66.7–95.3	2.25 (0.24–29.35)	0.6382
Discussion: Implications	20	27a	35.5 (11/31)	21.1–53.1	0.0 (0/22)	0.0–14.9	n.e.	n.e.
	20	27b	35.5 (11/31)	21.1–53.1	27.3 (6/22)	13.2–48.2	1.47 (0.45–4.83)	0.7396
	20	27c	35.5 (11/31)	21.1–53.1	54.6 (12/22)	34.7–73.1	0.46 (0.15–1.40)	0.2720

^a^Risk groups item was applicable only to 14 out of the 31 conventional studies; ^b^Class imbalance item was applicable only to 1 out of 22 machine learning-based studies

*P-values are presented for exploratory comparison of individual TRIPOD/TRIPOD+AI items. Each item represents a distinct reporting construct; therefore, no correction for multiple testing was applied

Adherence to Title and Abstract reporting was poor across both TRIPOD and TRIPOD + AI studies. Notably, no study fully adhered to the abstract reporting requirements, and many failed to report essential details such as the number of participants and outcome events. Title adherence was higher in TRIPOD studies (29.0%) than TRIPOD + AI (13.6%), though this difference was not statistically significant (OR: 2.55; 95CI%: 0.53–16.74).

Significant differences in adherence were observed in several key methodological domains. For example, reporting on the source of data (TRIPOD Item 4a vs. TRIPOD + AI Item 5a) was substantially better in TRIPOD studies (90.3%) compared to TRIPOD + AI studies (22.7%), (OR = 28.64, 95% CI: 5.65–212.03).

However, predictor selection and pre-processing (TRIPOD 10b vs. TRIPOD + AI 9a) was less thoroughly reported in TRIPOD studies (6.5%) than in TRIPOD + AI studies (50.0%), (OR = 0.07, 95% CI: 0.01–0.41). Furthermore, adherence to items detailing predictor definitions, measurements, and blinding was extremely poor across both frameworks, with no meaningful difference between the two.

Several areas demonstrated complete non-adherence. Not a single study provided a justification or calculation for sample size (TRIPOD Item 8; TRIPOD + AI Item 10). Likewise, model performance stability reporting, as outlined in TRIPOD + AI Item 23a, had 0.0% adherence, with machine learning studies failing to report on performance variability or bootstrap-based model evaluations. Protocol registration and access (TRIPOD + AI Item 18c) also showed 0.0% adherence.

In the interpretation of findings, there was a striking discrepancy: while 96.8% of TRIPOD studies adhered to Item 19b, none of the TRIPOD + AI studies met the requirements of the corresponding Item 25. Similarly, performance metric reporting (TRIPOD 10 d vs. TRIPOD + AI 12e) revealed a substantial gap. Most machine learning studies failed to report performance metrics at all evaluated time points.

Encouragingly, some areas showed stronger performance. Reporting of study limitations was generally high in both groups. Ethical approval reporting (TRIPOD + AI Item 17) was also well adhered to (96%). TRIPOD + AI studies also demonstrated full adherence (100%) to class imbalance reporting (Item 13).

### Total adherence to TRIPOD and TRIPOD + AI statements

**Fig. 2:**
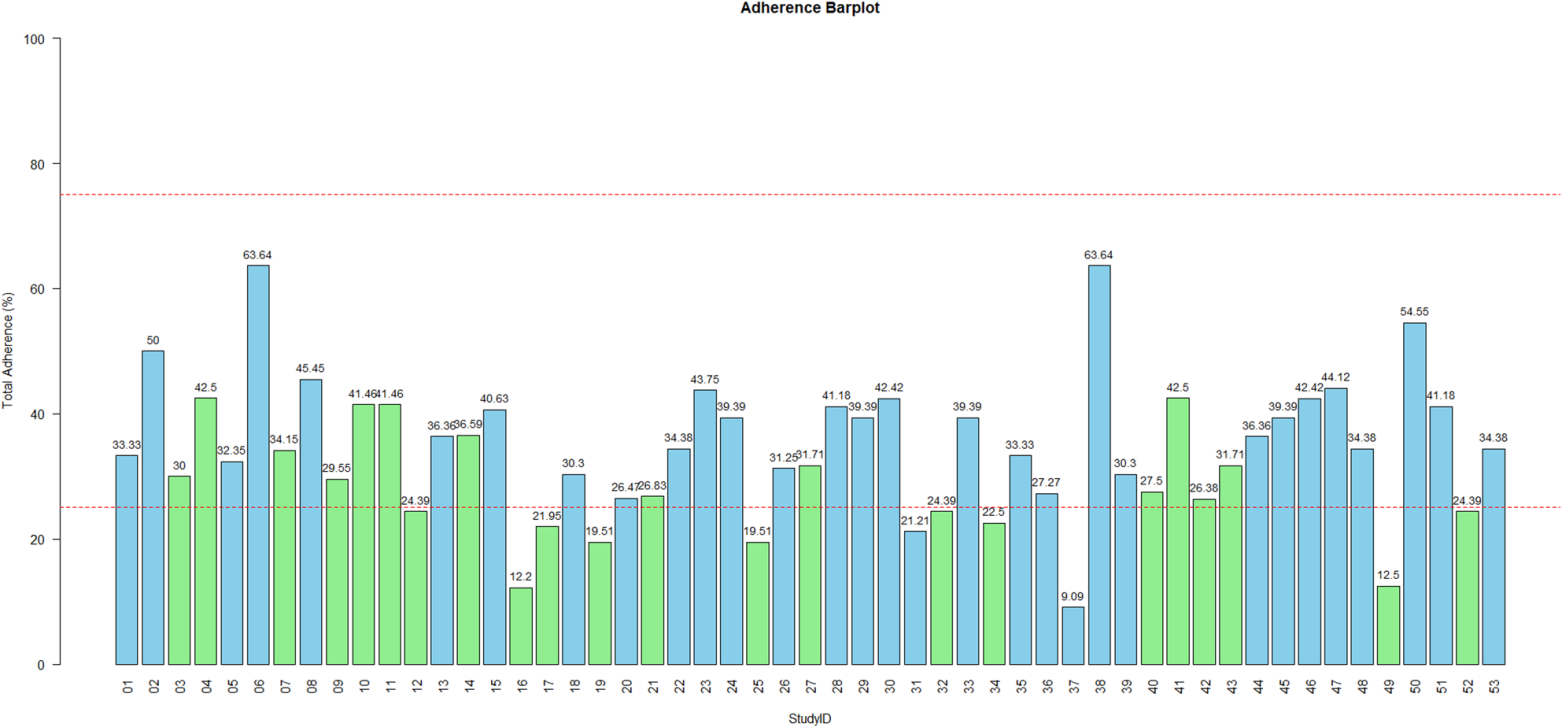
Barplot of total adherence to the TRIPOD (blue bars) and TRIPOD + AI (green bars) statements (red horizontal dashed lines represent 25% and 75% adherence, respectively)

Figure 2 shows the overall adherence of the included studies to the TRIPOD and TRIPOD + AI reporting guidelines illustrated in a bar chart. The overall mean adherence across all studies was 34.07% (SD: 11.15). Studies using conventional statistical models had a higher mean adherence to TRIPOD (38.11%, SD:10.39) compared to studies employing machine learning approaches, which showed lower adherence to TRIPOD + AI (28.37%, SD: 8.91; *p* = 0.0012).

## Discussion

This review provides a comprehensive assessment of reporting practices in 53 studies that developed and validated 71 COVID-19 prognostic models, offering insights into how these models were reported during a period of intense global research activity. Of these, 36 models (from 31 studies) used traditional statistical approaches, while 35 models (from 22 studies) relied on machine learning algorithms. Adherence to reporting guidelines for COVID-19 prognostic models was generally suboptimal, with several areas demonstrating significant deficiencies, particularly among machine learning-based studies. Although most models demonstrated moderate to excellent predictive performance, overall compliance with reporting standards was poor with average adherence being below 35%. Conventional models achieved a mean adherence of 38.1%, whereas machine learning–based studies lagged behind at 28.4%. This discrepancy is unsurprising given that TRIPOD + AI was only released in April 2024, two years after our latest included study.

Reporting weaknesses were most apparent in areas such as data preprocessing, clear definitions of predictors and outcomes, strategies for handling missing data, and justification of sample size. Such omissions obscure the risk of overfitting. Moreover, the limited sample sizes and low event counts in many studies heighten that risk. Inadequate detail on missing-data methods, lack of optimism correction in performance estimates, and insufficient calibration and validation approaches further undermine confidence in the reported C-index values, which often appear unrealistically high. Moreover, less than half (42.3%) of the studies made their final model fully available, with only 12.9% meeting TRIPOD minimums and a mere 4.6% satisfying TRIPOD + AI requirements. This lack of transparency poses serious barriers to replication and external validation.

These findings align with previous reviews showing poor adherence to TRIPOD guidelines in COVID-19 and other biomedical fields. They mirror earlier meta-research showing substandard TRIPOD adherence in COVID-19 prognostic modeling. Wynants et al.. highlighted widespread reporting deficiencies and high bias risk across COVID-19 prediction studies [10]. Yang et al.. reported TRIPOD completeness between 31% and 83% in COVID-19 models [20], while Dhiman et al.. found a median adherence of 41% in oncology machine learning models [12], and Liu et al.. observed 46.4% adherence in obstetric prognostic models [21]. Similarly, Navarro et al. [11]. found that reporting quality and risk of bias were suboptimal across machine learning–based clinical prediction models, reinforcing concerns about the transparency of such studies even beyond the COVID-19 context. The lower adherence observed in our study compared to previous reviews may be partly explained by our stricter inclusion criteria, which focused only on studies that reported development, internal validation, and external validation, thus excluding many earlier-stage or partial modeling studies included in previous reviews. Moreover, the application of the TRIPOD + AI update, which introduces more specific reporting expectations for AI-based models, could also contributed to lower adherence values than previously reported. Noncompliance with TRIPOD and TRIPOD + AI may also stem from misusing these tools as methodological guidelines instead of reporting standards. Furthermore, the urgency and volume of COVID-19 research contributed to rushed publications with inadequate reporting.

To improve the reliability and usability of prediction models, whether for COVID-19 or other conditions, researchers must rigorously apply TRIPOD or TRIPOD + AI in their reporting. Journal editors and peer reviewers should insist on these guidelines: in our sample, only 7.5% of studies appeared in journals that explicitly recommended TRIPOD, and just 20% of top-tier medical journals mention it in their author instructions [22].

This study employed a comprehensive search strategy and adhered to a pre-registered protocol (OSF: https://osf.io/kg9yw). A key strength was the integration of TRIPOD + AI, which addresses the unique reporting needs of machine learning-based models.

## Conclusions

Adherence to the TRIPOD and TRIPOD + AI reporting guidelines among COVID-19 prognostic prediction models was found to be notably low, particularly in the Methods and Results sections, which are critical for reproducibility, and was significantly worse among machine learning-based models (TRIPOD + AI) compared to conventional statistical models (TRIPOD) (*p* < 0.001). This poor reporting compromises the ability to assess model validity, limits clinical applicability, and often prevents external validation, at times making it unclear which predictors were included in the final model.

Raising awareness among researchers about the importance of complete and transparent reporting is essential, continued meta-research, and stronger editorial policies are needed to enforce compliance. Additionally, reporting guidelines themselves should be refined by moving beyond expert opinion to incorporate structured methodological frameworks. To address these issues, future studies should consistently follow TRIPOD + AI guidelines, and journals and peer reviewers should take a more active role in enforcing their use.

## Supplementary Information

Supplementary Material 1.

## Data Availability

The datasets supporting the conclusions of this article are available in the OSF Open Science Framework repository at [https://osf.io/kg9yw].
