# Supplementary material for: Machine learning-based COVID-19 prognostic models lag behind in reporting quality: findings from a TRIPOD/TRIPOD + AI systematic review
Source: Diagn Progn Res. 2026 Feb 3;10:3. doi: 10.1186/s41512-026-00218-x (PMC12866346; doi:10.1186/s41512-026-00218-x)
Supplement: Supplementary file 1 — Supplementary Material 1. [file 41512_2026_218_MOESM1_ESM.docx]

**Appendix Table 1.** Checklist of the PRISMA 2020 statement.

| **Section and Topic** | **Item #** | **Checklist item** | **Location where item is reported** |
| --- | --- | --- | --- |
| **TITLE** | | |  |
| Title | 1 | Identify the report as a systematic review. | Pg. 1 |
| **ABSTRACT** | | |  |
| Abstract | 2 | See the PRISMA 2020 for Abstracts checklist. | Pg. 2 |
| **INTRODUCTION** | | |  |
| Rationale | 3 | Describe the rationale for the review in the context of existing knowledge. | Pgs. 3 - 4 |
| Objectives | 4 | Provide an explicit statement of the objective(s) or question(s) the review addresses. | Pg. 3 - 4 |
| **METHODS** | | |  |
| Eligibility criteria | 5 | Specify the inclusion and exclusion criteria for the review and how studies were grouped for the syntheses. | Pg. 4 |
| Information sources | 6 | Specify all databases, registers, websites, organisations, reference lists and other sources searched or consulted to identify studies. Specify the date when each source was last searched or consulted. | Pg. 4 |
| Search strategy | 7 | Present the full search strategies for all databases, registers and websites, including any filters and limits used. | Pg. 4  (Ap. Table 2) |
| Selection process | 8 | Specify the methods used to decide whether a study met the inclusion criteria of the review, including how many reviewers screened each record and each report retrieved, whether they worked independently, and if applicable, details of automation tools used in the process. | Pgs. 4 - 5 |
| Data collection process | 9 | Specify the methods used to collect data from reports, including how many reviewers collected data from each report, whether they worked independently, any processes for obtaining or confirming data from study investigators, and if applicable, details of automation tools used in the process. | Pgs. 4 - 5 |
| Data items | 10a | List and define all outcomes for which data were sought. Specify whether all results that were compatible with each outcome domain in each study were sought (e.g. for all measures, time points, analyses), and if not, the methods used to decide which results to collect. | Pgs. 4 - 5 |
|  | 10b | List and define all other variables for which data were sought (e.g. participant and intervention characteristics, funding sources). Describe any assumptions made about any missing or unclear information. | Pgs. 4 - 5 |
| Study risk of bias assessment | 11 | Specify the methods used to assess risk of bias in the included studies, including details of the tool(s) used, how many reviewers assessed each study and whether they worked independently, and if applicable, details of automation tools used in the process. | N/A |
| Effect measures | 12 | Specify for each outcome the effect measure(s) (e.g. risk ratio, mean difference) used in the synthesis or presentation of results. | Pg. 5 |
| Synthesis methods | 13a | Describe the processes used to decide which studies were eligible for each synthesis (e.g. tabulating the study intervention characteristics and comparing against the planned groups for each synthesis (item #5)). | N/A |
|  | 13b | Describe any methods required to prepare the data for presentation or synthesis, such as handling of missing summary statistics, or data conversions. | N/A |
|  | 13c | Describe any methods used to tabulate or visually display results of individual studies and syntheses. | Pg. 5 |
|  | 13d | Describe any methods used to synthesize results and provide a rationale for the choice(s). If meta-analysis was performed, describe the model(s), method(s) to identify the presence and extent of statistical heterogeneity, and software package(s) used. | Pg. 5 |
|  | 13e | Describe any methods used to explore possible causes of heterogeneity among study results (e.g. subgroup analysis, meta-regression). | N/A |
|  | 13f | Describe any sensitivity analyses conducted to assess robustness of the synthesized results. | N/A |
| Reporting bias assessment | 14 | Describe any methods used to assess risk of bias due to missing results in a synthesis (arising from reporting biases). | N/A |
| Certainty assessment | 15 | Describe any methods used to assess certainty (or confidence) in the body of evidence for an outcome. | N/A |
| **RESULTS** | | |  |
| Study selection | 16a | Describe the results of the search and selection process, from the number of records identified in the search to the number of studies included in the review, ideally using a flow diagram. | Pgs. 5 - 6 |
|  | 16b | Cite studies that might appear to meet the inclusion criteria, but which were excluded, and explain why they were excluded. | N/A |
| Study characteristics | 17 | Cite each included study and present its characteristics. | Pg. 6  (Ap. Table 4) |
| Risk of bias in studies | 18 | Present assessments of risk of bias for each included study. | N/A |
| Results of individual studies | 19 | For all outcomes, present, for each study: (a) summary statistics for each group (where appropriate) and (b) an effect estimate and its precision (e.g. confidence/credible interval), ideally using structured tables or plots. | Pg. 6  (Ap. Table 6) |
| Results of syntheses | 20a | For each synthesis, briefly summarise the characteristics and risk of bias among contributing studies. | N/A |
|  | 20b | Present results of all statistical syntheses conducted. If meta-analysis was done, present for each the summary estimate and its precision (e.g. confidence/credible interval) and measures of statistical heterogeneity. If comparing groups, describe the direction of the effect. | Pgs. 6 – 7  (Ap. Table 6) |
|  | 20c | Present results of all investigations of possible causes of heterogeneity among study results. | N/A |
|  | 20d | Present results of all sensitivity analyses conducted to assess the robustness of the synthesized results. | N/A |
| Reporting biases | 21 | Present assessments of risk of bias due to missing results (arising from reporting biases) for each synthesis assessed. | N/A |
| Certainty of evidence | 22 | Present assessments of certainty (or confidence) in the body of evidence for each outcome assessed. | N/A |
| **DISCUSSION** | | |  |
| Discussion | 23a | Provide a general interpretation of the results in the context of other evidence. | Pgs. 7 - 8 |
|  | 23b | Discuss any limitations of the evidence included in the review. | Pg. 8 |
|  | 23c | Discuss any limitations of the review processes used. | Pg. 8 |
|  | 23d | Discuss implications of the results for practice, policy, and future research. | Pg. 8 |
| **OTHER INFORMATION** | | |  |
| Registration and protocol | 24a | Provide registration information for the review, including register name and registration number, or state that the review was not registered. | Pgs. 2, 4, 9 |
|  | 24b | Indicate where the review protocol can be accessed, or state that a protocol was not prepared. | Pgs. 2, 4, 9 |
|  | 24c | Describe and explain any amendments to information provided at registration or in the protocol. | N/A |
| Support | 25 | Describe sources of financial or non-financial support for the review, and the role of the funders or sponsors in the review. | Pg. 9 |
| Competing interests | 26 | Declare any competing interests of review authors. | Pg. 9 |
| Availability of data, code and other materials | 27 | Report which of the following are publicly available and where they can be found: template data collection forms; data extracted from included studies; data used for all analyses; analytic code; any other materials used in the review. | Pg. 9 |

**Appendix Table 2.** Search algorithm for the different databases (search date: July 2024).

| **MEDLINE** |
| --- |
| ((“severe acute respiratory syndrome coronavirus 2”[Supplementary Concept] OR “COVID-19” [Supplementary Concept] OR “coronavirus” OR “corona virus” OR “HCoV” OR “nCoV” OR “2019 CoV” OR “covid” OR “covid19” OR “Severe Acute Respiratory Syndrome Coronavirus 2” OR “SARS-CoV2” OR “SARS-CoV 2” OR “SARS Coronavirus 2”) AND (“systematic review” OR “review” OR “meta-analysis”) AND ((Validat$ OR Predict$.ti. OR Rule$) OR (Predict$ AND (Outcome$ OR Risk$ OR Model$)) OR ((History OR Variable$ OR Criteria OR Scor$ OR Characteristic$ OR Finding$ OR Factor$) AND (Predict$ OR Model$ OR Decision$ OR Identif$ OR Prognos$)) OR (Decision$ AND (Model$ OR Clinical$ OR Logistic Models/)) OR (Prognostic AND (History OR Variable$ OR Criteria OR Scor$ OR Characteristic$ OR Finding$ OR Factor$ OR Model$)))) AND (("2019/11/01"[Date - Publication] : "2022/12/31"[Date - Publication])) |
| **Epistemonikos.org** |
| (title:((title:(systematic review) OR abstract:(systematic review)) AND (title:(prognostic model) OR abstract:(prognostic model)) AND (title:(covid-19) OR abstract:(covid-19))) OR abstract:((title:(systematic review) OR abstract:(systematic review)) AND (title:(prediction model) OR abstract:(prediction model)) AND (title:(covid-19) OR abstract:(covid-19)))) |
| **Scopus** |
| ( ( "severe acute respiratory syndrome coronavirus 2" OR covid-19 OR coronavirus OR "corona virus" OR Hcov OR nCoV OR "2019 CoV" OR covid OR covid19 OR "Severe Acute Respiratory Syndrome Coronavirus 2" OR sars-cov2 OR "SARS-CoV 2" OR "SARS Coronavirus 2" ) AND ( "systematic review" OR review OR meta-analysis ) AND ( ( validat? OR predict? OR rule? ) OR ( predict? AND ( outcome? OR risk? OR model? ) ) OR ( ( history OR variable? OR criteria OR scor? OR characteristic? OR finding? OR factor? ) AND ( predict? OR model? OR decision? OR identif? OR prognos? ) ) OR ( decision? AND ( model? OR clinical? OR "Logistic Models" ) ) OR ( prognostic AND ( history OR variable? OR criteria OR scor? OR characteristic? OR finding? OR factor? OR model? ) ) ) ) AND PUBYEAR > 2019 AND PUBYEAR < 2023 |
| **Cochrane Database of Systematic Reviews (CDSR)** |
| ((“severe acute respiratory syndrome coronavirus 2” OR “COVID-19” OR “coronavirus” OR “corona virus” OR “HCoV” OR “nCoV” OR “2019 CoV” OR “covid” OR “covid19” OR “Severe Acute Respiratory Syndrome Coronavirus 2” OR “SARS-CoV2” OR “SARS-CoV 2” OR “SARS Coronavirus 2”) AND (“systematic review” OR “review” OR “meta-analysis”) AND ((Validation OR Prediction OR Rules) OR (Prediction AND (Outcomes OR Risk OR Models)) OR ((History OR Variables OR Criteria OR Scores OR Characteristics OR Findings OR Factors) AND (Prediction OR Models OR Decision OR Identification OR Prognosis)) OR (Decision AND (Model OR Clinical OR Logistic Models)) OR (Prognostic AND (History OR Variables OR Criteria OR Scores OR Characteristics OR Findings OR Factors OR Models)))) |

**Appendix Table 3.** Extracted items list from each eligible study.

| **Related Characteristic** | **Information Extracted** |
| --- | --- |
| Target Population/Setting | i) Single country data (yes/no), ii) Country (specify country), iii) Dataset (Hospital/Repository name), iv) Study dates (start and end dates), v) Care setting (hospitalized patients, outpatients, etc.), vi) Status of target population (Covid-19 cases, symptomatic, asymptomatic, etc.), v) Study design (prospective, retrospective cohort, etc.). |
| Participants | i) Recruitment method, ii) Number of centers involved, iii) Inclusion/exclusion criteria, iv) participant description (age and sex). |
| Sample Size | i) Number of participants and number of outcomes/events, ii) Number of outcomes/events in relation to the number of candidate predictors (Events Per Variable). |
| Missing Data Handling | i) Number of participants with missing values, ii) Number of participants with missing data for each predictor, iii) Handling of missing data (e.g., complete-case analysis, imputation, or other methods). |
| Study Description/Model Development | i) Modelling method, ii) Method for selection of predictors in multivariable model, iii) Method for selection during multivariable analysis. |
| Model Performance | i) Calibration plot, ii) C-statistic, iii) Specificity, iv) Sensitivity, etc. |
| Internal Validation | i) Method for internal validation, ii) Shrinkage of predictor weights or regression coefficients, iii) Number of participants for internal validation and number of participants with outcome, iv) Performance measures (C-statistic, Sensitivity, Specificity, etc.), v) Model adjustments or update. |
| External Validation | i) External validation dataset (centers, country, recruitment method etc.), ii) Number of participants for external validation and number of participants with outcome, iii) Performance measures (C-statistic, Sensitivity, Specificity, etc.), iv) Model adjustments or update. |
| Final Model | i) Final model presented with intercept and predictor weights, ii) Tools for model presentation (nomogram, partial regression formula, etc.), iii) Available URL to access model, software object, model code, etc. |
| Transparency evaluation | The following transparency indicators were assessed on study or journal level: i) Protocol registration, ii) Protocol availability, iii) Data sharing statement, iv) Data availability statement, v) Access to raw data, vi) Code availability, vii) Data Sharing Policy, viii) Funding statement, ix) Conflict of interest statement and x) Journal policy on authors guidelines. |

**Appendix Table 4.** List of the seventeen (17) systematic reviews.

|  | **First Author** | **Title** | **Pub. Year** | **No. of cited studies** |
| --- | --- | --- | --- | --- |
| 01 | Adamidi ES | Artificial intelligence in clinical care amidst COVID-19 pandemic: A systematic review | 2021 | 100 |
| 02 | Alballa N | Machine learning approaches in COVID-19 diagnosis, mortality, and severity risk prediction: A review | 2021 | 50 |
| 03 | Appel KS | A Systematic Review of Predictor Composition, Outcomes, Risk of Bias, and Validation of COVID-19 Prognostic Scores | 2023 | 192 |
| 04 | Bottino F | COVID Mortality Prediction with Machine Learning Methods:  A Systematic Review and Critical Appraisal | 2021 | 24 |
| 05 | Buttia C | Prognostic models in COVID‑19 infection that predict severity:  a systematic review | 2023 | 309 |
| 06 | Cardenas-Fuentes G | Validity of prognostic models of critical COVID-19 is variable. A  systematic review with external validation | 2023 | 14 |
| 07 | Chee ML | Artificial Intelligence Applications for COVID-19 in Intensive Care and Emergency Settings: A Systematic Review | 2021 | 14 |
| 08 | Chen R | Prediction of prognosis in COVID-19 patients using machine learning: A systematic review and meta-analysis | 2023 | 33 |
| 09 | Chu K | Evaluating risk stratification scoring systems to predict mortality in patients with COVID-19 | 2021 | 76 |
| 10 | Dabbagh R | Harnessing Machine Learning in Early COVID-19 Detection and Prognosis: A Comprehensive Systematic Review | 2023 | 66 |
| 11 | dos Santos AL | Machine learning algorithms to predict outcomes in children and  adolescents with COVID-19: a systematic review | 2023 | 9 |
| 12 | Miller JL | Prediction models for severe manifestations and mortality due  to COVID-19: A systematic review | 2021 | 78 |
| 13 | Montazeri M | Machine Learning Models for Image-Based Diagnosis and  Prognosis of COVID-19: Systematic Review | 2021 | 44 |
| 14 | Shakeel SM | COVID-19 prediction models: a systematic literature review | 2021 | 9 |
| 15 | Shakibfar S | Artificial intelligence-driven prediction of COVID-19-related  hospitalization and death: a systematic review | 2023 | 39 |
| 16 | Wang L | Artificial Intelligence for COVID-19: A Systematic Review | 2021 | 60 |
| 17 | Wynants L | Prediction models for diagnosis and prognosis of covid-19: systematic review and critical appraisal | 2022 | 412 |

**Appendix Table 5.** Development, internal validation and external validation prognostic studies included in the present study.

|  | **First Author** | **Title** | **Pub. Year** | **No. of models** | **Type of model** |
| --- | --- | --- | --- | --- | --- |
| 01 | Alhamar G | Development of a clinical risk score to predict death in patients with COVID-19 | 2022 | 1 | Conventional |
| 02 | Allenbach Y | Development of a multivariate prediction model of intensive care unit transfer or death: A French prospective cohort study of hospitalized COVID-19 patients | 2020 | 1 | Conventional |
| 03 | Aznar-Gimeno R | A clinical decision web to predict ICU admission or death for patients hospitalised with COVID-19 using machine learning algorithms | 2021 | 1 | Machine learning |
| 04 | Bertsimas D | COVID-19 mortality risk assessment: An international multi-center study | 2020 | 1 | Machine learning |
| 05 | Cai L | Predictive Nomogram for Severe COVID-19 and Identification of Mortality-Related Immune Features | 2021 | 1 | Conventional |
| 06 | Carr E | Evaluation and improvement of the National Early Warning Score (NEWS2) for COVID-19: a multi-hospital study | 2021 | 1 | Conventional |
| 07 | Chowdhury MEH | An Early Warning Tool for Predicting Mortality Risk of COVID‑19 Patients Using Machine Learning | 2020 | 1 | Machine learning |
| 08 | Fan X | Scores based on neutrophil percentage and LDH with or without SaO2 predict hospital mortality risk in severe COVID-19 patients | 2021 | 2 | Conventional |
| 09 | Feng Z | Machine learning based on clinical characteristics and chest CT quantitative measurements for prediction of adverse clinical outcomes in hospitalized patients with COVID-19 | 2021 | 1 | Machine learning |
| 10 | Gao Y | Machine learning based early warning system enables accurate mortality risk prediction for COVID-19 | 2020 | 1 | Machine learning |
| 11 | Gao Y | Development and validation of an online model to predict critical COVID-19 with immune-inflammatory parameters | 2021 | 1 | Machine learning |
| 12 | Guan X | Clinical and inflammatory features based machine learning model for fatal risk prediction of hospitalized covid-19 patients: results from a retrospective cohort study | 2021 | 1 | Machine learning |
| 13 | Hajifathalian K | Development and external validation of a prediction risk model for short-term mortality among hospitalized U.S. COVID-19 patients: A proposal for the COVID-AID risk tool | 2020 | 2 | Conventional |

**Appendix Table 5.** *Continued*.

|  | **First Author** | **Title** | **Pub. Year** | **No. of models** | **Type of model** |
| --- | --- | --- | --- | --- | --- |
| 14 | He F | The development and validation of simplified machine learning algorithms to predict prognosis of hospitalized patients With COVID-19: multicenter, retrospective study | 2022 | 4 | Machine learning |
| 15 | Heber S | A model predicting mortality of hospitalized Covid-19 patients four days after admission: development, internal and temporal-external validation | 2021 | 1 | Conventional |
| 16 | Hiremath A | Integrated clinical and CT based artificial intelligence nomogram for predicting severity and need for ventilator support in COVID-19 patients: a multi-site study | 2021 | 1 | Machine learning |
| 17 | Hu C | Early prediction of mortality risk among patients with severe COVID-19, using machine learning | 2021 | 1 | Machine learning |
| 18 | Jamal MH | A biomarker based severity progression indicator for COVID-19: the Kuwait prognosis indicator score | 2020 | 1 | Machine learning |
| 19 | Jimenez-Solem E | Developing and Validating Covid-19 Adverse Outcome Risk Prediction Models From a Bi-National European Cohort of 5594 Patients | 2021 | 3 | Machine learning |
| 20 | Lasbleiz A | Phenotypic characteristics and development of a hospitalization prediction risk score for outpatients with diabetes and covid-19: The diabcovid study | 2020 | 1 | Conventional |
| 21 | Lassau N | Integrating deep learning CT-scan model, biological and clinical variables to predict severity of COVID-19 patients | 2021 | 1 | Machine learning |
| 22 | Levy TJ | Development and Validation of a Survival Calculator for Hospitalized Patients with COVID-19 | 2020 | 1 | Conventional |
| 23 | Li J | Derivation and validation of a prognostic model for predicting in-hospital mortality in patients admitted with COVID-19 in Wuhan, China: the PLANS (platelet lymphocyte age neutrophil sex) model | 2020 | 1 | Conventional |
| 24 | Li L | Development and validation of a prognostic nomogram for predicting in-hospital mortality of COVID-19: a multicenter retrospective cohort study of 4086 cases in China | 2021 | 1 | Conventional |
| 25 | Li S | Development and external evaluation of predictions models for mortality of covid-19 patients using machine learning method | 2023 | 1 | Machine learning |
| 26 | Liang W | Development and validation of a clinical risk score to predict the occurrence of critical illness in hospitalized patients with covid-19 | 2020 | 1 | Conventional |

**Appendix Table 5.** *Continued*.

|  | **First Author** | **Title** | **Pub. Year** | **No. of models** | **Type of model** |
| --- | --- | --- | --- | --- | --- |
| 27 | Liang W | Early triage of critically ill COVID-19 patients using deep learning | 2020 | 1 | Machine learning |
| 28 | Liu H | Development and validation of a risk score using complete blood count to predict in-hospital mortality in COVID19 patients | 2021 | 1 | Conventional |
| 29 | Liu J | Development and validation of a prediction model for early identification of critically ill elderly COVID-19 patients | 2020 | 1 | Conventional |
| 30 | Liu L | A simple nomogram for predicting failure of non-invasive respiratory strategies in adults with COVID-19: a retrospective multicentre study | 2021 | 1 | Conventional |
| 31 | Liu L | Early prediction model for progression and prognosis of severe patients with coronavirus disease 2019 | 2021 | 1 | Conventional |
| 32 | Ma X | Characteristic of 523 COVID-19 in Henan Province and a Death Prediction Model | 2020 | 1 | Machine learning |
| 33 | Magro B | Predicting in-hospital mortality from coronavirus disease 2019: A simple validated app for clinical use | 2021 | 1 | Conventional |
| 34 | Marcos M | Development of a severity of disease score and classification model by machine learning for hospitalized COVID-19 patients | 2021 | 1 | Machine learning |
| 35 | Martin S | Development and validation of a laboratory-based risk score to predict the occurrence of critical illness in hospitalized patients with COVID-19 | 2021 | 1 | Conventional |
| 36 | McRae MP | Clinical decision support tool and rapid point-of-care platform for determining disease severity in patients with COVID-19 | 2020 | 1 | Conventional |
| 37 | McRae MP | Managing COVID-19 With a Clinical Decision Support Tool in a Community Health Network: Algorithm Development and Validation | 2020 | 2 | Conventional |
| 38 | Mei J | Development and external validation of a COVID-19 mortality risk prediction algorithm: A multicentre retrospective cohort study | 2020 | 2 | Conventional |
| 39 | Razavian N | A validated, real-time prediction model for favorable outcomes in hospitalized COVID-19 patients | 2020 | 1 | Conventional |
| 40 | Ryan L | Mortality prediction model for the triage of COVID-19, pneumonia, and mechanically ventilated ICU patients: A retrospective study | 2020 | 1 | Machine learning |

**Appendix Table 5.** *Continued*.

|  | **First Author** | **Title** | **Pub. Year** | **No. of models** | **Type of model** |
| --- | --- | --- | --- | --- | --- |
| 41 | Schoning V | Development and validation of a prognostic COVID-19 severity assessment (COSA) score and machine learning models for patient triage at a tertiary hospital | 2021 | 1 | Machine learning |
| 42 | Shashikumar P | Development and Prospective Validation of a Deep Learning Algorithm for Predicting Need for Mechanical Ventilation | 2021 | 1 | Machine learning |
| 43 | Vaid A | Machine learning to predict mortality and critical events in a cohort of patients with COVID-19 in New York City: Model development and validation | 2020 | 8 | Machine learning |
| 44 | Weng Z | ANDC: An early warning score to predict mortality risk for patients with Coronavirus Disease 2019 | 2020 | 1 | Conventional |
| 45 | Wu G | Development of a clinical decision support system for severity risk prediction and triage of COVID-19 patients at hospital admission: An international multicentre study | 2020 | 1 | Conventional |
| 46 | Xia Y | A nomogram to early predict isolation length for non-severe COVID-19 patients based on laboratory investigation: A multicenter retrospective study in Zhejiang Province, China | 2021 | 1 | Conventional |
| 47 | Xiao LS | Development and validation of the HNC-LL score for predicting the severity of coronavirus disease 2019 | 2020 | 1 | Conventional |
| 48 | Xie J | Development and external validation of a prognostic multivariable model on admission for hospitalized patients with COVID-19 | 2020 | 1 | Conventional |
| 49 | Yao Z | Construction and validation of a machine learning‐based nomogram: A tool to predict the risk of getting severe coronavirus disease 2019 (COVID‐19) | 2021 | 1 | Machine learning |
| 50 | Zhang B | Clinical utility of a nomogram for predicting 30-days poor outcome in hospitalized patients with COVID-19: multicenter external validation and decision curve analysis | 2020 | 1 | Conventional |
| 51 | Zhang H | Risk prediction for poor outcome and death in hospital in-patients with COVID-19: derivation in Wuhan, China and external validation in London, UK | 2020 | 2 | Conventional |
| 52 | Zheng Y | A Learning-Based Model to Evaluate Hospitalization Priority in COVID-19 Pandemics | 2020 | 1 | Machine learning |
| 53 | Zheng Y | Development and validation of a prognostic nomogram based on clinical and CT features for adverse outcome prediction in patients with COVID-19 | 2020 | 1 | Conventional |

**Appendix Table 6.** Methodological characteristics of the included models.

| Characteristics on Model Level (n = 71) | |
| --- | --- |
| Participants for Model Development, median (IQR) | 679 (275 – 1552) |
| Not reported, N (%) | 0 (0) |
|  |  |
| Participants with Outcome for Model Development, median (IQR) | 112 (48 – 363) |
| Not reported, N (%) | 6 (8.5) |
|  |  |
| Candidate Predictors for Model Development, *median (IQR)* | 42.00 (21.50 – 51.25) |
| Not reported, N (%) | 15 (21.1) |
|  | |
| Events per Candidate Predictor for Model Development, *median (IQR)* | 3.71 (1.40 – 12.40) |
| Not reported, N (%) | 21 (29.6) |
|  | |
| Handling of Missing Data | N (%) |
| Single imputation | 19 (26.8) |
| Multiple imputation | 14 (19.7) |
| Other | 13 (18.3) |
| Not reported | 12 (16.9) |
| Complete-case analysis | 10 (14.1) |
| Indicator methods | 3 (4.2) |
|  | |
| Method of Modelling | N (%) |
| Logistic regression | 36 (50.7) |
| Tree based | 23 (32.4) |
| Cox proportional hazards regression | 4 (5.6) |
| Neural networks | 4 (5.6) |
| Other | 4 (5.6) |
|  | |
| Method for Predictors Selection in Multivariable Model | N (%) |
| All candidate predictors | 38 (53.5) |
| Preselection based on univariable analysis | 13 (18.3) |
| Other | 11 (15.5) |
| Not reported / Unclear | 9 (12.7) |
|  | |
| Method for Predictors Selection During Multivariable Analysis | N (%) |
| Other | 25 (35.2) |
| LASSO | 16 (22.5) |
| Backward (stepwise) selection | 10 (14.1) |
| Added value | 8 (11.3) |
| All candidate predictors | 4 (5.6) |
| Not applicable | 4 (5.6) |
| Unclear | 3 (4.2) |
| Forward (stepwise) selection | 1 (1.4) |
|  | |
| Calibration Plot (Development) | N (%) |
| Presented | 22 (31.0) |
| Not presented | 49 (69.0) |
|  | |

**Appendix Table 6.** *Continued*.

| Characteristics on Model Level (n = 71) | |
| --- | --- |
| C-statistic (Concordance Index) (Development), median (IQR) | 0.86 (0.82 – 0.94) |
| Not reported, N (%) | 31 (43.7) |
|  |  |
| Sensitivity (Development), %, median (IQR) | 75.00 (66.00 – 80.00) |
|  |  |
| Specificity (Development), *%, median (IQR)* | 86.00 (79.00 – 92.00) |
| Not reported, N (%) | 50 (70.4) |
|  | |
| Internal Validation Method | N (%) |
| Cross-validation (x-fold) | 27 (38.0) |
| Random split | 17 (23.9) |
| Resampling (e.g., bootstrapping) | 12 (16.9) |
| Temporal split | 12 (16.9) |
| Other | 3 (4.2) |
|  | |
| Shrinkage of Predictor Weights or Regression Coefficients | N (%) |
| Not reported | 51 (71.8) |
| Lasso/Ridge | 10 (14.1) |
| Calibration slope assessed with bootstrapping | 6 (8.5) |
| Unclear | 2 (2.8) |
| Heuristic shrinkage | 1 (1.4) |
| Penalized maximum likelihood estimation | 1 (1.4) |
|  | |
| Participants for Internal Validation, *median (IQR)* | 383 (167 – 654) |
| Not reported, N (%) | 33 (46.5) |
|  |  |
| Participants with Outcome for Internal Validation, *median (IQR)* | 29 (15 – 165) |
| Not reported, N (%) | 43 (60.6) |
|  | |
| Calibration Plot (Internal Validation) | N (%) |
| Presented | 37 (52.1) |
| Not presented | 34 (47.9) |
|  | |
| C-statistic (Concordance Index) (Internal Validation), *mean (SD)* | 0.86 (15.63) |
| Not reported, N (%) | 8 (11.3) |
|  |  |
| Sensitivity (Internal Validation), *%, median (IQR)* | 73.50 (53.50 – 89.00) |
| Not reported, N (%) | 41 (57.7) |
|  | |
| Specificity (Internal Validation), *%, median (IQR)* | 85.00 (77.00 – 89.00) |
| Not reported, N (%) | 42 (59.2) |
|  |  |
| Participants for External Validation, *median (IQR)* | 286 (122 – 1048) |
| Not reported, N (%) | 0 (0) |
|  | |
| Participants with Outcome for External Validation, *median (IQR)* | 60 (21 – 333) |
| Not reported, N (%) | 6 (8.5) |
|  | |

**Appendix Table 6.** *Continued*.

| Characteristics on Model Level (n = 71) | |
| --- | --- |
| Dataset Type for External Validation | N (%) |
| New centers, same country | 40 (56.3) |
| New centers, different country | 17 (23.9) |
| Other | 14 (19.7) |
|  |  |
| Study Design (External Validation) | N (%) |
| Retrospective | 38 (53.5) |
| Prospective | 17 (23.9) |
| Unclear | 11 (15.5) |
| Registry Data | 4 (5.6) |
| Other | 1 (1.4) |
|  | |
| Recruitment Method (External Validation) | N (%) |
| Unclear | 43 (60.6) |
| Consecutive | 28 (39.4) |
|  | |
| Calibration Plot (External Validation) | N (%) |
| Presented | 48 (67.6) |
| Not presented | 23 (32.4) |
|  | |
| C-statistic (Concordance Index) (External Validation), *mean (SD)* | 0.84 (0.09) |
| Not reported, N (%) | 4 (5.6) |
|  | |
| Sensitivity (External Validation), *%, median (IQR)* | 76.00 (67.00 – 86.25) |
| Not reported, N (%) | 39 (54.9) |
|  | |
| Specificity (External Validation), *%, median (IQR)* | 82.00 (73.00 – 92.25) |
| Not reported, N (%) | 39 (54.9) |
|  | |
| Final Model Presented | N (%) |
| Yes | 30 (42.3) |
| No | 41 (57.7) |
|  | |
| Tools for Model Presentation | N (%) |
| Online tool | 25 (35.2) |
| Nomogram | 18 (25.3) |
| Not presented | 18 (25.3) |
| Sum score | 7 (9.9) |
| Mobile app | 2 (2.8) |
| Software object | 1 (1.4) |
|  | |
| URL to Access Model, Software object, Model Code | N (%) |
| Not applicable | 31 (43.7) |
| URL presented and is working | 22 (31.0) |
| URL is no longer working/responding | 18 (25.3) |
